# Mortality and Cancer in Offspring of Mothers With Biopsy‐Proven MASLD During Pregnancy: A Nationwide Cohort Study

**DOI:** 10.1111/liv.70174

**Published:** 2025-06-11

**Authors:** Carole A. Marxer, Fahim Ebrahimi, David Bergman, Jiangwei Sun, Hannes Hagström, Marcus Thuresson, Olof Stephansson, Jonas F. Ludvigsson

**Affiliations:** ^1^ Department of Medical Epidemiology and Biostatistics Karolinska Institutet Stockholm Sweden; ^2^ Department of Gastroenterology and Hepatology University Digestive Health Care Center Basel ‐ Clarunis Basel Switzerland; ^3^ Division of Hepatology, Department of Upper GI Diseases Karolinska University Hospital Stockholm Sweden; ^4^ Department of Medicine Huddinge, Karolinska Institutet Stockholm Sweden; ^5^ Statisticon AB Uppsala Sweden; ^6^ Department of Medicine Solna, Karolinska Institutet Stockholm Sweden; ^7^ Department of Women's Health, Division of Obstetrics Karolinska University Hospital Stockholm Sweden; ^8^ Department of Paediatrics Örebro University Hospital Örebro Sweden; ^9^ Department of Medicine Columbia University College of Physicians and Surgeons New York New York USA

**Keywords:** cancer, epidemiology, MASLD, mortality, NAFLD, paediatrics

## Abstract

**Background & Aims:**

Health‐related outcomes through early adulthood among offspring prenatally exposed to maternal metabolic dysfunction‐associated steatotic liver disease (MASLD) are insufficiently investigated. We aimed to study the risk of mortality and cancer in such offspring.

**Methods:**

This nationwide cohort study included all singleton live born offspring with prenatal exposure to maternal biopsy‐proven MASLD (1992–2017; *N* = 239) in Sweden. MASLD offspring were matched with up to five reference offspring (*N* = 1131) of mothers without known MASLD by maternal age at delivery, calendar year of delivery, and parity. We used a multivariable Cox proportional hazard model to calculate adjusted hazard ratios (aHRs) with 95% confidence intervals (CIs) for mortality and cancer up until 31 December 2021. For mortality, we stratified by maternal MASLD severity (simple steatosis alone vs. severe MASLD comprising steatohepatitis, liver fibrosis or cirrhosis).

**Results:**

Over a median follow‐up of 16.9 years, two deaths occurred in offspring prenatally exposed to maternal MASLD (IR 0.5/1000 person‐years, 95% CI 0.1–1.8) and seven deaths in reference offspring (IR 0.4/1000 person‐years, 95% CI 0.1–0.8), which corresponded to an aHR of 1.78 (95% CI 0.27–11.97). Higher disease severity was not associated with an increased risk of death. We observed few cancer events with similar IR/1000 person‐years (0.2 [95% CI 0.0–1.4] vs. 0.3 [0.1–0.6] in reference offspring), which corresponded to an aHR of 0.64 (95% CI 0.07–5.95).

**Conclusions:**

We found no evidence that prenatal exposure to maternal MASLD affects the risk of death or cancer through early adulthood but larger studies are needed.

**Summary**

- Around 10% of women of childbearing age are affected by metabolic dysfunction‐associated steatotic liver disease (MASLD), which can lead to complications during pregnancy and childbirth; however, the extent to which MASLD in pregnant women influences the health of the offspring remains unclear.
- In this study, we assessed the most severe potential health problems in newborns of mothers with MASLD between the date of birth and early adulthood.
- We found no evidence that MASLD during pregnancy increases the risk of death or cancer in offspring through early adulthood compared to children of mothers without MASLD, but larger studies are needed to confirm our findings.

**Abbreviations**
aHR: adjusted hazard ratio
BMI: body mass index
CI: confidence interval
ESPRESSO: Epidemiology Strengthened by histopathology Reports in Sweden
HR: hazard ratio
ICD: international classification of diseases
IQR: interquartile range
LISA: Longitudinal Integrated Database for Health Insurance and Labour Market Studies
MASH: metabolic dysfunction‐associated steatohepatitis
MASLD: metabolic dysfunction‐associated steatotic liver disease
MBR: Medical Birth Register
NAFLD: non‐alcoholic fatty liver disease
NPR: National Patient Register
PDR: Prescribed Drug Register
PPV: positive predictive value
US: United States

## 1 Introduction

Metabolic dysfunction‐associated steatotic liver disease (MASLD)—formerly known as non‐alcoholic fatty liver disease (NAFLD) [1]—is the most common chronic liver disease worldwide, with an estimated prevalence of 38% [2] (23% in middle‐aged adults in Sweden [3]). In women of childbearing age (20–40 years of age), the prevalence is estimated to be around 10% [4], and depends on factors such as the prevalence of obesity, type 2 diabetes, and other metabolic disorders in the examined population [5], as well as age, mode of diagnosis (ultrasound, non‐invasive tests, and/or liver biopsy), and access to diagnostic modalities [6].

Prior observational studies have collectively reported that MASLD in pregnant women may lead to a meaningful increase in adverse materno‐fetal outcomes such as preterm birth, gestational diabetes, and gestational hypertension towards the end of pregnancy, some of them independent of obesity [7, 8, 9, 10, 11, 12, 13, 14].

However, prior studies assessing outcomes of in utero exposure to MASLD beyond birth are—to our knowledge—limited to two studies, both with short follow‐up periods [14, 15]. First, a hospital‐based study (2019–2022) from the United States (US) prospectively evaluated weight, growth, and neonatal outcomes in 14 offspring born to mothers with MASLD, compared to 80 offspring born to mothers without MASLD, over their first 2 years. The study did not find an increased risk of neonatal death (< 28 days after date of birth) associated with MASLD [15]. Second, our previous Swedish observational study (1992–2017) suggests that MASLD does not influence the risk of neonatal death [14]. Evidence on other adverse effects in offspring prenatally exposed to maternal MASLD—such as mortality beyond 28 days and cancer—remains, to the best of our knowledge, nonexistent. However, such knowledge has important healthcare implications given the high global prevalence of MASLD in women of childbearing age and its significant impact on both mothers and their offspring.

Our previous Swedish observational study has shown that maternal MASLD increases the risk of preterm birth by more than 3‐fold, potentially contributing to higher mortality rates among affected offspring [14]. Further, a large observational study from the United Kingdom [16] showed that obesity (a strong risk factor of MASLD) was associated with a 35% increased risk of premature death in 1550 adult offspring compared to 27 051 offspring of mothers with a normal body mass index (BMI). However, if MASLD itself increases the risk of offspring mortality is so far unknown. Similar risk increases have been shown also for cancer among offspring of obese mothers. For example, a study from the US has shown that offspring born to mothers with obesity had a 1.32‐fold (95% CI 1.08–1.62) increased risk of any cancer and a 1.57‐fold (95% CI 1.12–2.20) increased risk of leukaemia compared to offspring born to mothers with a normal BMI. As for mortality, it is unknown if MASLD itself also increases the risk of cancer and if yes, if the risk increase is independent of maternal obesity and other metabolic disorders in the mother [17].

We aimed to leverage the nationwide matched ESPRESSO (Epidemiology Strengthened by histopathology Reports in Sweden) cohort to investigate all‐cause mortality and incident cancer through early adulthood in offspring born to mothers with biopsy‐proven MASLD versus offspring born to mothers without known MASLD or other liver diseases.

## 2 Methods

### 2.1 Data Sources

We used data from the ESPRESSO [18] cohort between 1992 and 2021. This cohort contains liver biopsy data from all pathology departments in Sweden (*n* = 28) linked to nationwide Swedish healthcare registers [18, 19]. Linkage was performed using the unique personal identity number assigned to all Swedish residents at the time of birth or immigration to Sweden [19]. We used the *Medical Birth Register* (*MBR*), which started in 1973, and covers 99% of all births in Sweden. This register contains data on maternal conditions during pregnancy as well as clinical characteristics and lifestyle factors such as BMI and smoking (BMI recorded since 1992, smoking recorded since 1982) [20]. The *MBR* prospectively collects maternal data from the beginning of the first prenatal visit which usually takes place before the 12th gestational week [20]. We retrieved data on maternal comorbidities from the *National Patient Register (NPR)* which contains data on diagnoses and procedures in non‐primary healthcare since 1964 [21]. In 1987, the *NPR* became nationwide, and in 2001, data on diagnoses recorded during non‐primary outpatient visits were added [21]. In addition to the *NPR*, we used the *Prescribed Drug Register* (*PDR*) to define certain maternal comorbidities based on medication information. The *PDR* contains information on medications dispensed at pharmacies in Sweden since 2005 (July 1) [22]. Data on maternal education was retrieved from the Swedish *Longitudinal Integrated Database for Health Insurance and Labour Market Studies* (LISA) [23]. Registers used to extract outcome data in offspring are described in section *Outcomes*.

### 2.2 Study Design

We performed a nationwide matched cohort study and followed offspring from the date of birth +1 day (index date) until the date of emigration from Sweden, administrative censoring (31 December 2021), or until the occurrence of an outcome (death or cancer, in separate analyses). The index date was set to 1 day after the date of birth to not mistakenly include stillborn offspring. A graphical illustration of the longitudinal study design is depicted in Figure S1.

### 2.3 Study Population

Our study population built on a previous study which evaluated the risk of pregnancy and birth outcomes among singleton offspring live born or stillborn to women with versus without MASLD [14].

#### 2.3.1 Offspring of Women With MASLD

We identified singleton offspring of women with MASLD between 15 and 44 years of age at delivery through the *MBR*. MASLD was identified by the first liver biopsy histopathology report that included a SNOMED topography code for liver (T56) and a SNOMED morphology code for steatosis (M5008x or M5520x) between 1 January 1965 and 31 December 2017 (index liver biopsy). To enhance the specificity of our identification of MASLD, we used a validated *International Classification of Diseases* (ICD)‐based algorithm, which follows international expert panel consensus recommendations [24] (positive predictive value [PPV] = 92% [25]). This algorithm considers the exclusion of (1) individuals with a recorded ICD code for other concomitant chronic liver conditions prior to the *delivery date* (e.g., recorded other aetiology of liver disease, prior alcohol abuse, liver transplantation; Table S1) and (2) individuals who had emigrated from Sweden before the date of the index liver biopsy. We defined the date of MASLD diagnosis as the date of the index liver biopsy, which required to be prior to the date of delivery of the offspring of interest.

We used SNOMED definitions [26] to identify histological subgroups of MASLD. Since MASLD is a progressive disease, we defined the histological subgroup based on the most recent histopathology report with MASLD before the date of delivery of the offspring of interest. Histological subgroups included simple steatosis, MASH without fibrosis, MASLD with noncirrhotic fibrosis, or cirrhosis due to MASLD (definitions in Table S2) [25]. We pooled all individuals with MASH with or without fibrosis and cirrhosis (‘severe MASLD’) to perform stratified analyses.

#### 2.3.2 Reference Offspring of Mothers Without MASLD

As comparators, we used singleton live born offspring of reference women without known MASLD and without any other liver disease (definitions in Table S1) between 15 and 44 years of age at delivery.

#### 2.3.3 Matching of Offspring

**FIGURE 1:**
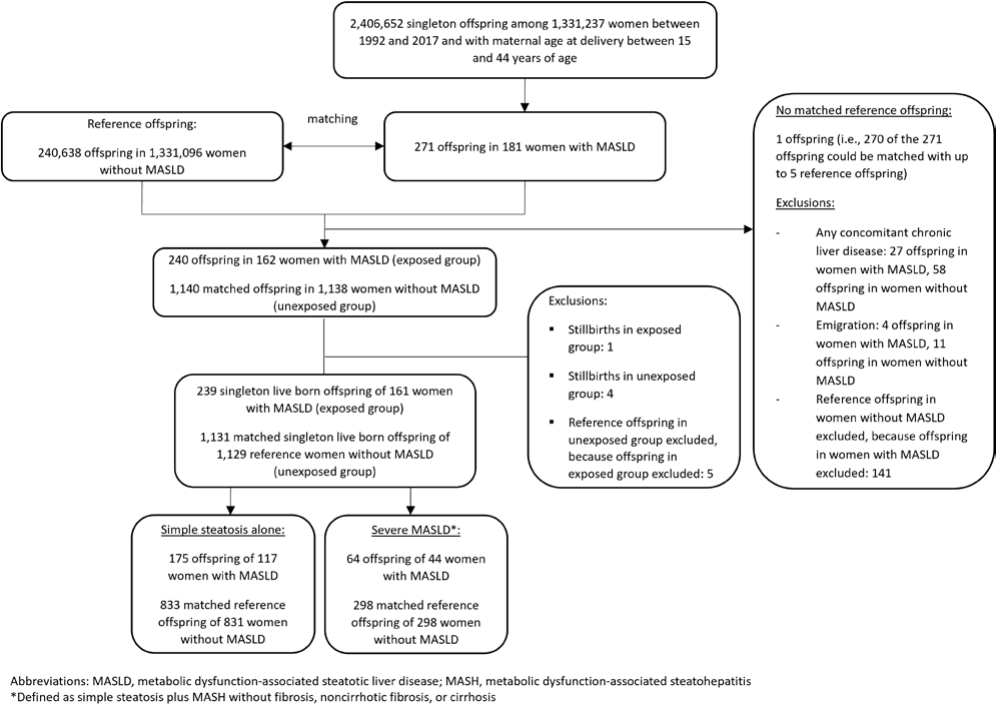
Flow chart of cohort enrolment.

The observation unit was a singleton live born offspring, and a woman could contribute several offspring to the study population. We matched each singleton live born offspring of a woman with MASLD with up to five singleton live born offspring of reference women according to maternal age at delivery, calendar year of delivery, and parity (Figure 1).

#### 2.3.4 Exclusion of Stillborn Offspring

After matching, we excluded stillborn offspring in both groups (definition of stillbirth in Table S3). In case of stillbirth among offspring of women with MASLD, we excluded all corresponding matches (up to 5).

### 2.4 Outcomes

We studied all‐cause mortality and incident cancer during follow‐up. We obtained data on mortality from the Swedish *Cause of Death Register* [27], which covers all deaths in Sweden since 1952 [27]. Due to the small number of events, we did not distinguish between different causes of death. We retrieved incident cancer diagnoses from the Swedish *National Cancer Register* (founded in 1958 [28]) based on ICD codes (Table S4).

### 2.5 Statistical Analyses

We used descriptive statistics to present demographics and baseline characteristics (Table S5). We calculated incidence rates (IRs) per 1000 person‐years with 95% confidence intervals (CIs) for the outcomes mortality and cancer during follow‐up compared with reference offspring.

We estimated multivariable‐adjusted hazard ratios (HRs) and 95% CIs for the outcomes using Cox proportional hazards regression models. We presented HRs (a) conditioned on the matching set including maternal age at delivery, calendar year of delivery, and parity (model 1), and (b) additionally adjusted for maternal obesity‐related covariates, namely BMI in early pregnancy, diabetes, and pre‐eclampsia (model 2). *Post hoc*, we performed a sensitivity analysis to evaluate if including level of education (proxy for socioeconomic status) as an additional covariate in model 2 would change the results (model 3).

Variables such as birth weight, gestational age at birth, caesarean delivery, as well as onset of MASLD or other metabolic disorders in the offspring were left out, as these could act as mediators in the pathway between maternal MASLD and offspring outcomes. However, to account for preterm birth as a potential mediator in the association between prenatal exposure to MASLD exposure and offspring mortality, we conducted a sensitivity analysis by restricting our study population to term‐born offspring (≥ 37 gestational weeks). In a *post hoc* sensitivity analysis, we also evaluated if SGA may act as a potential mediator in the association between maternal MASLD and offspring mortality by restricting our study population to offspring with normal weight for gestational age.

In sub‐analyses, we examined mortality by histological subgroups of MASLD, namely simple steatosis versus severe MASLD. In a *post hoc* sensitivity analysis, we restricted our analyses to the first‐born offspring in both the exposed and unexposed groups. We did not perform secondary analyses for cancer due to data privacy concerns (fewer events for cancer than for mortality).

We performed all statistical analyses using R version 4.3 (R Foundation for Statistical Computing, Vienna, Austria).

### 2.6 Ethics

This project was approved by the Stockholm Ethics Review Board. Due to the register‐based nature of the study, informed consent was waived. The Ethics Review Board requested that data were presented in an aggregate form, and hence we were not allowed to present the characteristics of individual patients.

## 3 Results

### 3.1 Study Cohort

We identified 239 live born offspring (45.4% female) to 161 women with MASLD and 1131 matched reference offspring (46.7% female) of 1129 women without MASLD and other liver diseases (Figure 1). Compared to reference offspring, a higher proportion of offspring of mothers with MASLD were born preterm (16.7% vs. 4.6%), had a low birth weight (10.9% vs. 3.4%), were small for gestational age (SGA; 14.7% vs. 8.6%), large for gestational age (LGA; 20.2% vs. 12.1%), and were delivered via caesarean section (32.2% vs. 16.0%; Table 1). The median maternal age at delivery was 32 years (interquartile range [IQR] 27–36) and 39% of pregnancies were nulliparous. Mothers with MASLD, compared to mothers without known MASLD, were less educated (27% vs. 45% with ≥ 13 years of education), smoked more frequently during early pregnancy (17% vs. 10%), were more frequently obese (39% vs. 10%), and had higher rates of prior diabetes (10.5% vs. 0.9%), hypertension (5.0% vs. 0.5%), dyslipidemia (1.7% vs. 0.2%), and pre‐eclampsia (7.9% vs. 3.1%; Table 1).

**TABLE 1:** Baseline characteristics of offspring with in utero exposure to maternal MASLD and matched reference offspring.

	Offspring of mothers with MASLD	Reference offspring
Offspring, *n*	239	1131
Unique mothers, *n*	161	1129
Years of follow‐up		
Median (IQR)	16.9 [11.6, 22.1]	16.8 [11.5, 22.3]
< 18	134 (56.1)	644 (56.9)
≥ 18	105 (43.9)	487 (43.1)
Offspring characteristics		
Female sex	108 (45.4)	528 (46.7)
Calendar year of date of birth (i.e., start of follow‐up)		
1992–1999	64 (26.8)	308 (27.2)
2000–2010	125 (52.3)	595 (52.6)
2011–2017	50 (20.9)	228 (20.2)
Gestational age at birth [days], median (IQR)	274.0 [264.0, 283.0]	281.0 [274.0, 287.0]
Preterm birth		
Any preterm birth (< 37 weeks)	40 (16.7)	52 (4.6)
Medically indicated	23 (9.6)	14 (1.2)
Spontaneous	17 (7.1)	37 (3.3)
Very preterm (< 32 weeks)	7 (2.9)	9 (0.8)
Fetal growth		
Birth weight [g]		
Median (IQR)	3493 [2984, 3895]	3600 [3249, 3930]
Low (< 2500)	26 (10.9)	38 (3.4)
Normal (2500 to < 4000)	166 (69.5)	853 (75.4)
High (≥ 4000)	46 (19.2)	236 (20.9)
Missing	1 (0.4)	4 (0.4)
Small for gestational age (SGA)	35 (14.7)	97 (8.6)
Large for gestational age (LGA)	48 (20.2)	136 (12.1)
Apgar < 7 at 5 min	4 (1.7)	15 (1.3)
Congenital malformations	16 (6.7)	61 (5.4)
Delivered by		
Induction of labor	49 (21.1)	156 (13.9)
Caesarean section	77 (32.2)	181 (16.0)
Instrumental delivery	15 (6.3)	82 (7.3)
Maternal characteristics		
Maternal age at delivery [years]		
Median (IQR)	32.0 [27.0, 36.0]	32.0 [27.0, 36.0]
15 to < 25	26 (10.9)	123 (10.9)
25 to < 35	134 (56.1)	637 (56.3)
35 to 44	79 (33.1)	371 (32.8)
Liver histology of maternal MASLD		
Simple steatosis	175 (73.2)	—
MASH without fibrosis	30 (12.6)	—
Noncirrhotic fibrosis	30 (12.6)	—
Cirrhosis	4 (1.7)	—
Year of maternal MASLD diagnosis (index liver biopsy)		
Up until 1999	161 (67.4)	—
2000–2010	71 (29.7)	—
2011–2017	7 (2.9)	—
Disease duration (time between first MASLD diagnosis and delivery [years]		
Median (IQR)	5.6 [3.1, 9.9]	—
< 5	102 (42.7)	—
5 to < 10	80 (33.5)	—
≥ 10	57 (23.8)	—
Maternal country of birth		
Nordic	203 (84.9)	936 (82.8)
Other	36 (15.1)	195 (17.2)
Civil status of the mother		
Living with partner	207 (86.6)	1018 (90.0)
Not living with partner	11 (4.6)	21 (1.9)
Missing	21 (8.8)	92 (8.1)
Education		
Compulsory school (≤ 9 years)	36 (15.1)	116 (10.3)
Upper secondary school (10–12 years)	140 (58.6)	485 (42.9)
College or university (≥ 13 years)	63 (26.4)	508 (44.9)
Missing	0 (0.0)	22 (1.9)
Parity: multiparous	147 (61.5)	695 (61.5)
BMI in early pregnancy [kg/m^2^]		
Median (IQR)	28.7 [25.0, 33.2]	23.9 [21.5, 26.8]
< 18.5	0 (0.0)	23 (2.0)
18.5 to < 25	55 (23.0)	585 (51.7)
25 to < 30	72 (30.1)	271 (24.0)
≥ 30	92 (38.5)	116 (10.3)
Missing	20 (8.4)	136 (12.0)
Smoking in early pregnancy		
Yes	41 (17.2)	114 (10.1)
No	187 (78.2)	959 (84.8)
Missing	11 (4.6)	58 (5.1)
Prior comorbidities and conditions		
Diabetes^a^	25 (10.5)	10 (0.9)
Hypertension^b^	12 (5.0)	6 (0.5)
Dyslipidemia	4 (1.7)	2 (0.2)
Pre‐eclampsia	19 (7.9)	35 (3.1)

*Note:* Values are *n* (%), unless otherwise indicated.

Abbreviations: BMI, body mass index; IQR, interquartile range; MASH, metabolic dysfunction‐associated steatohepatitis; MASLD, metabolic dysfunction‐associated steatotic liver disease; n, number; SD, standard deviation.

^a^ Diabetes type 1, diabetes type 2, or gestational diabetes.

^b^ Including gestational hypertension.

Among mothers with MASLD, 73% (*n* = 175) had liver histopathology indicating simple steatosis alone, while 27% (*n* = 64) had severe MASLD. Further baseline characteristics by disease severity are listed in Table S6. Baseline characteristics of term born offspring and their mothers are listed in Table S7 (sensitivity analysis).

### 3.2 Mortality

#### 3.2.1 Main Analysis

During a median follow‐up of 16.9 (IQR 11.6–22.1) years, two offspring born to mothers with MASLD died (IR 0.5 per 1000 person‐years, 95% CI 0.1–1.8) at a mean age of 0.3 years (SD 0.3; Table 2, Table 3). During a similar median follow‐up in reference offspring (16.8 years, IQR 11.5–22.3), seven offspring died (IR 0.4 per 1000 person‐years, 95% CI 0.1–0.8) at a mean age of 7.8 years (SD 10.7; Table 2, Table 3). After multivariable adjustment (model 2), the risk of death among offspring of mothers with MASLD and reference offspring was not increased (HR 1.78, 95% CI 0.27–11.97; Table 3). Results remained consistent after additionally adjusting for level of education (HR 1.47, 95% CI 0.23–9.32; Table 3). The point estimate increased when restricting our analyses to the first‐born offspring within each woman; however, the 95% CI overlapped with that of the main analysis and included 1 (HR 2.70 [95% CI 0.34–21.56] vs. HR 1.78 [95% CI 0.27–11.97] in main analysis; Table S9).

**TABLE 2:** Characteristics of deaths and cancer diagnoses.

	Offspring of mothers with MASLD	Reference offspring
Offspring, *n*	239	1131
Unique mothers, *n*	161	1129
All‐cause mortality		
Death during follow‐up, *n*	2	7
Age at death [years], mean (SD)	0.3 (0.3)	7.8 (10.7)
Incident cancer		
Cancer during follow‐up, *n*	NR^a^	NR^a^
Age at cancer diagnosis [years], mean (SD)	NR^a^	22.3 (6.6)

*Note:* Values are *n* (%), unless otherwise indicated.

Abbreviations: MASLD, metabolic dysfunction‐associated steatotic liver disease; *n*, number; NR, not reported; SD, standard deviation.

^a^ Not reported (NR) due to data privacy concerns. In total, we identified six cancer events.

**TABLE 3:** All‐cause mortality and incident cancer through early adulthood among offspring with in utero exposure to maternal MASLD and matched reference offspring.

	*N*	Events	py	IR per 1000 py (95% CI)	Unadjusted HR (95% CI) Model 1^a^	Adjusted HR (95% CI) Model 2^b^	Adjusted HR (95% CI) Model 3 (*post hoc* sensitivity analysis)^c^
All‐cause mortality							
Overall	1370	9	23 048	0.4 (0.2–0.7)			
Reference offspring	1131	7	19 007	0.4 (0.1–0.8)	1 (Reference)	1 (Reference)	1 (Reference)
Offspring of mothers with MASLD	239	2	4041	0.5 (0.1–1.8)	1.36 (0.28–6.56)	1.78 (0.27–11.97)	1.47 (0.23–9.32)
Incident cancer							
Overall	1370	6	23 027	0.3 (0.1–0.6)			
Reference offspring	1131	NR^d^	18 998	0.3 (0.1–0.6)	1 (Reference)	1 (Reference)	1 (Reference)
Offspring of mothers with MASLD	239	NR^d^	4029	0.2 (0.0–1.4)	0.91 (0.11–7.79)	0.64 (0.07–5.95)	1.01 (0.13–7.52)

Abbreviations: CI, confidence interval; HR, hazard ratio; IR, incidence rate; MASLD, metabolic dysfunction‐associated steatotic liver disease; py, person‐years.

^a^ Model 1: conditioned on matching set.

^b^ Model 2: conditioned on matching set and further adjusted for maternal obesity‐related factors BMI in early pregnancy, diabetes (including gestational diabetes) any time prior to delivery, and pre‐eclampsia any time prior to delivery.

^c^ Model 3: same as model 2, but additionally adjusted for level of education (post hoc sensitivity analysis).

^d^ Not reported (NR) due to data privacy concerns. In total, we identified six cancer events.

#### 3.2.2 Maternal MASLD Severity

Stratified by histological severity of maternal MASLD, we did not observe higher death rates in offspring of mothers with severe MASLD compared to offspring of mothers with simple steatosis alone (IR 0.6 per 1000 person‐years; 95% CI 0.1–2.3; Tables S8 and S9). After multivariable adjustment (model 2), none of the maternal MASLD severity levels were associated with offspring mortality (simple steatosis alone: HR 3.59, 95% CI 0.48–26.79, severe MASLD: HR could not be calculated due to lack of deaths among offspring to mothers in this stratum; Tables S8 and S9).

#### 3.2.3 Mortality in Term Born Offspring

There were no deaths among term born offspring of mothers with MASLD, while four of the total seven deaths among reference offspring occurred in term born offspring (Tables S8 and S9).

#### 3.2.4 Mortality in Offspring With Normal Birth Weight for Gestational Age

There were no deaths among offspring with normal birth weight for gestational age born to mothers with MASLD, while five of a total of seven deaths among reference offspring occurred in offspring with normal birth weight for gestational age (Tables S8 and S9).

### 3.3 Cancer

During follow‐up, there were fewer cancer events compared to mortality, with six incident cancer events among offspring of mothers with MASLD or reference offspring (no reporting of number of events by exposure group due to data privacy concerns). The IR/1000 person‐years (95% CI) was similar (0.2 [95% CI 0.0–1.4] vs. 0.3 [0.1–0.6] in reference offspring), which corresponded to an adjusted HR of 0.64 (95% CI 0.07–5.95; model 2; Table 2, Table 3). Results remained consistent after additionally adjusting for level of education (HR 1.01, 95% CI 0.13–7.52; Table 3).

## 4 Discussion

### 4.1 Summary

This nationwide cohort study reports for the first time the risk of all‐cause mortality and incident cancer through early adulthood among offspring with prenatal exposure to maternal biopsy‐confirmed MASLD compared with reference offspring to mothers without known MASLD or other liver diseases. During a median follow‐up of 16.9 years, 1.26% of 239 MASLD offspring and 1.06% of 1131 reference offspring died or developed cancer. After adjusting for confounders, we found no association between prenatal MASLD exposure and the risk of death or cancer in offspring. The mortality findings remained consistent (i.e., no risk increase) when restricting to offspring of mothers with severe MASLD, but statistical power was limited in this analysis. Our findings should comfort affected individuals and physicians and are especially important given the high and rising global prevalence of MASLD among women of childbearing age (10% [4]) and the absence of previous studies.

### 4.2 Validity of Results

The lack of excess mortality and cancer through early adulthood in offspring born to mothers with MASLD suggests that maternal MASLD during pregnancy does not confer an obvious increased risk of death or cancer in their offspring. This conclusion remained when restricting to the first birth of a woman (in contrast to our main analyses in which a woman could contribute several offspring to the study population). However, given the limited statistical power, these findings should be interpreted with caution.

The following two arguments reinforce the validity of our results: First, it is reassuring that we did not observe a significant association, even though our study may overrepresent women with more complex or severe MASLD due to the requirement of a biopsy‐proven diagnosis for inclusion. While most patients with MASLD are asymptomatic [29], we assume that women with a biopsy‐proven diagnosis tend to be sicker on average, with more comorbidities and complications, which results in more healthcare visits and a higher chance of diagnosing MASLD. More complex MASLD and/or a higher maternal disease burden at baseline likely lead to worse outcomes in offspring but was not observed in our study. This indicates that prenatal exposure to MASLD likely does not increase the risk of mortality and cancer in offspring. Second, it is reassuring that the death rate among offspring did not increase with maternal MASLD severity, as one would expect if there were a positive association with mortality in offspring. Both deaths were observed in offspring with prenatal exposure to simple steatosis alone (73% of the study population), while no death occurred in offspring with prenatal exposure to severe MASLD (27%). Of note, MASLD is a progressive disease, and increasing disease severity may reduce fertility, potentially resulting in a smaller proportion of offspring born to mothers with severe MASLD. This, in turn, limits the interpretation of outcomes among offspring of mothers with severe MASLD. The two deaths observed among offspring born to women with simple steatosis alone might suggest that women with severe MASLD receive closer monitoring in clinical practice during pregnancy, leading to better care and, consequently, fewer adverse outcomes in their offspring. However, larger studies are needed for further exploration.

### 4.3 Inexistence of Previous Literature

While several studies exist on pregnancy and birth outcomes among births of women with MASLD [8, 9, 12, 13, 14], no previous studies have evaluated offspring mortality and incident cancer through early adulthood. However, two prior studies investigated the risk of neonatal death, and none found any association: The first study evaluated the rate of neonatal death among 94 offspring of mothers with vs. without MASLD during pregnancy born between 2019 and 2022 at a single US hospital [15]. The second study is our previous registry‐based study in Sweden (1992–2017), which examined 240 offspring of women with MASLD, compared to 1140 matched births from women without MASLD [14].

### 4.4 Preterm Birth and SGA: Mediators of Infant Death?

We found that among offspring with prenatal MASLD exposure, the mean age at death was meaningfully lower compared to reference offspring (0.3 vs. 7.8 years). We cannot rule out an increased mortality in the first year of life among MASLD offspring compared to reference offspring, but it is more likely that preterm birth and potentially SGA resulted in premature death. Several studies have shown that maternal MASLD is associated with a significantly increased risk of preterm birth [8, 9, 12, 13, 14]. Preterm birth and especially very preterm birth can lead to adverse outcomes across the life span of an offspring [30], and may potentially mediate the association between maternal MASLD and adverse outcomes in the offspring. However, our attempt to assess if preterm birth is a potential mediator was limited by statistical power. The association between maternal MASLD and SGA in the offspring is less prominent. Results from previous studies are conflicting and it might be possible that some mechanisms favour LGA [31], while others favour SGA [32]. Again, statistical power limits our attempt to assess if SGA acts as a mediator between maternal MASLD and offspring mortality. Thus, we cannot conclude if SGA acts as a mediator or if the result was obtained by chance.

### 4.5 Other Adverse Outcomes Among Offspring

It should be noted that this study focused on the most severe endpoints of prenatal MASLD exposure, and we cannot rule out other potentially harmful effects on growth and development in the offspring from maternal MASLD. Therefore, our findings do not implicate that offspring born to mothers with MASLD do not require additional surveillance throughout their life course. It is possible that these offspring experience a higher onset of comorbidities or morbidity over time, including the development of MASLD, obesity, type 2 diabetes, or other metabolic disorders. However, such studies have not yet been conducted. Future research should also examine whether the potential onset of comorbidities in these offspring increases the risk of long‐term mortality.

### 4.6 An Insufficiently Investigated Research Area

This study highlights the lack of evidence on health‐related outcomes among offspring born to mothers with MASLD. The limited availability of data sources to study offspring outcomes in women with MASLD is likely the reason for this underexplored area of research. However, data from other countries are important for studying population differences. For example, we cannot rule out that in countries where antenatal and perinatal healthcare is inadequate, prenatal exposure to maternal MASLD could influence the rate of adverse outcomes. Another likely reason for the limited investigation of adverse offspring outcomes following prenatal exposure to MASLD is the widespread underdiagnosis of MASLD due to the asymptomatic nature of the condition. It is estimated that 10% of women of childbearing age have MASLD [4], yet many are unaware of the condition because they do not experience noticeable symptoms [29]. Furthermore, not all routinely collected data allow for the linkage of maternal and offspring records to study the effects of prenatal exposures on offspring outcomes. Even when such linkages are possible, follow‐up is often limited, as most MASLD diagnoses have occurred in the past two decades due to the increasing prevalence and awareness of the condition.

### 4.7 Strengths and Limitations

This study has several strengths. First, it encompasses 99% of all births in Sweden, highlighting its population‐based nature. Second, the specificity of MASLD diagnosis based on liver histology was high (PPV = 92%), as well as the validity of covariates (PPV = 85%–95% for most diagnoses in the *NPR* [21]). Third, the underlying ESPRESSO cohort allowed us to stratify our analyses by maternal MASLD severity, which is unique to ESPRESSO. Fourth, our model included maternal BMI, which is reliably recorded in Swedish registers since the start of the study period in 1992 [20].

This study has the following limitations. First, statistical power was the main limitation of our study. To reach a statistical power of 80% at a significance level of 0.05 and given the underlying parameters (sample size etc.), we would need to observe an HR of at least 6.2 for mortality and 7.6 for cancer. Despite the low statistical power, it is important to provide clinicians with these results given that no previous study has assessed the same research question and given that evidence based on larger study populations is unlikely in the near future, since other pre‐existing data have important limitations (discussed earlier). Second, due to the small number of mortality and cancer events, we were not able to adjust our analyses for more confounders. Therefore, we cannot rule out residual confounding. However, obesity‐related factors are the most important confounders and were included in the multivariable adjusted model. Third, due to the few numbers of mortality and cancer events, we were not able to report causes of death and the type of cancer. Fourth, previous studies have shown variability across ethnicities in susceptibility to MASLD, but information on ethnicity was not available because Swedish registers do not collect this information. Therefore, our findings may not be generalisable to other ethnicities than Caucasian, which is the predominant population in Sweden.

## 5 Conclusion

This nationwide study found no evidence that prenatal exposure to maternal MASLD is associated with increased all‐cause mortality and cancer through early adulthood, but statistical power was limited. Our findings are comforting to affected individuals and physicians, and important due to the high and increasing global prevalence of MASLD in women of childbearing age. However, our findings do not implicate that offspring born to mothers with MASLD do not require additional surveillance throughout their life course. Future studies should evaluate the occurrence of other adverse outcomes in these offspring, and larger studies are needed to confirm our findings regarding long‐term risk of mortality and cancer.

## Author Contributions

**Carole A. Marxer:** conceptualisation, data curation, formal analysis, funding acquisition, investigation, methodology, software, project administration, validation, visualisation, writing – original draft preparation, writing – review and editing. **Fahim Ebrahimi:** conceptualisation, investigation, methodology, resources, validation, writing – review and editing. **David Bergman:** conceptualisation, investigation, methodology, validation, writing – review and editing. **Jiangwei Sun:** conceptualisation, investigation, methodology, validation, writing – review and editing. **Hannes Hagström:** conceptualisation, investigation, methodology, validation, writing – review and editing. **Marcus Thuresson:** data curation, investigation, methodology, software, validation, writing – review and editing. **Olof Stephansson:** conceptualisation, investigation, methodology, validation, writing – review and editing. **Jonas F. Ludvigsson:** conceptualisation, funding acquisition, investigation, methodology, project administration, resources, supervision, validation, writing – review and editing.

## Ethics Statement

This study was approved by the Ethics Review Board in Stockholm, Sweden (2014/1287–31/4, 2018/972–32 and 2022–05774‐02).

## Consent

Due to the register‐based nature of the study, informed consent was waived.

## Conflicts of Interest

F.E. has served as an advisory board member for Boehringer Ingelheim. H.H. institutions have received research funding from Astra Zeneca, EchoSens, Gilead, Intercept, MSD, Novo Nordisk and Pfizer. H.H. has served as consultant or on advisory boards for Astra Zeneca, Bristol Myers‐Squibb, MSD and Novo Nordisk and has been part of hepatic events adjudication committees for Arrowhead, Boehringer Ingelheim, KOWA and GW Pharma. J.F.L. Dr. Ludvigsson has coordinated an unrelated study on behalf of the Swedish IBD quality register (SWIBREG). That study received funding from Janssen corporation. Dr. Ludvigsson has also received financial support from Merck/MSD on a project about inflammatory bowel disease, and for developing a paper about national healthcare registers in China. Dr. Ludvigsson has an ongoing research collaboration on celiac disease with Takeda. All the other authors declare no conflicts of interest.

## Supporting information


Data S1.

## Data Availability

Data are not available because of Swedish data protection regulations.
