# Supplementary material for: Mortality and Cancer in Offspring of Mothers With Biopsy‐Proven MASLD During Pregnancy: A Nationwide Cohort Study
Source: Liver Int. 2025 Jun 11;45(7):e70174. doi: 10.1111/liv.70174 (PMC12153413; doi:10.1111/liv.70174)
Supplement: Supplementary file 1 — Data S1. [file LIV-45-0-s001.docx]

**SUPPLEMENTARY MATERIAL**

**Title:** Mortality and cancer in offspring of mothers with biopsy-proven MASLD during pregnancy: A nationwide cohort study

**Authors:** Carole A. Marxer, Fahim Ebrahimi, David Bergman, Jiangwei Sun, Hannes Hagström, Marcus Thuresson, Olof Stephansson, Jonas F. Ludvigsson


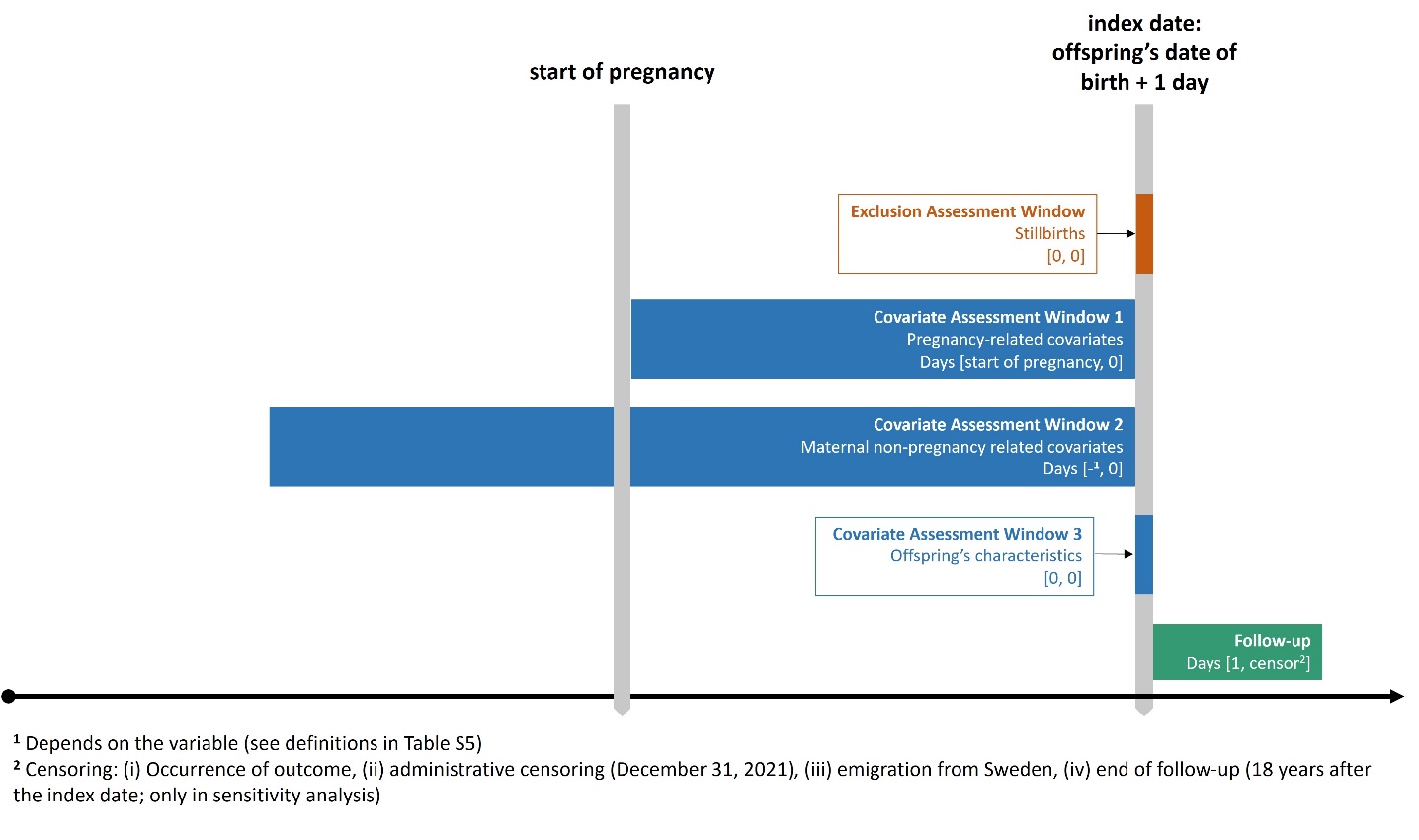


**Figure S1:** Study design.

**Table S1:** Exclusion of any concomitant chronic liver condition among women with MASLD and reference women.

| Excluded conditions^1^ | ICD-8 (1969-1986) | ICD-9 (1987-1996) | ICD-10 (1997-) |
| --- | --- | --- | --- |
| Alcohol abuse / misuse, or Alcohol-related liver disease | 280,00; 281,00; 307,00; 307,10; 307,99; 322; 581,10; 583,10; 261,00; 262,00; 291; 291,1; 303; 571,00; 571,01; 979; 980,00; 980,01; 980,98; 980,99 | 291; 294A; 303; 305A; 357F; 425F; 535D; 571A-D; 760W; 790D; 977D; 980A; 980X; V97B | E24.4; F10; G31.2; G62.1; G72.1; I42.6; K29.2; K70; K85.2; K86.0; Q35.4; R78.0; T51.0; T51.8; T51.9; X65; Y15; Y57.3; Y90; Y91; Z50.2; Z71.4; Z72.1 |
| Other abuse- and drug-related diagnoses | 571,0; E860; N980 | 571A-D | F11-F19 |
| Drug-induced liver disease | − | 573D | K71 |
| Viral hepatitis (e.g. hepatitis B, C) | 070; 999,20 | 070 | B15-19; B00.8; B25.1 |
| Budd-Chiari | − | 453A | I82 |
| Liver abscess | 572 | 572A | K75.0; A06.4 |
| HIV | 079,83; Y40,49; Y41,49 | 279K | B20-B24; F02.4; Z21.9; Z71.7 |
| Hemochromatosis | 273,2 | 275A | E83.1 |
| Wilson’s disease | 273,3 | 275B | E83.0 |
| Autoimmune hepatitis | − | 573D; 571E | K75.4 |
| Primary biliary cholangitis | − | 571G | K74.3; K74.4 |
| Other cholangitis | 574,06 | 576B | K83; K83.0A |
| Alpha-1 antitrypsin deficiency | − | 277G | E88.0 |
| Glycogen storage disease |  | 271W, 271X | E74 |
| Liver transplantation^†^ | − | V42H 5200-5299^†^ | Z94.4 JJC, DJ005; DJ006^†^ |
| Gastric bypass surgery | − | − | JDF^†^ |

The same table was presented in our previous study.^1^

Abbreviations: ICD, International Classification of Disease; HIV, human immunodeficiency virus

^†^Liver transplantation and bariatric surgery were further defined via procedure codes.

^1^ We will exclude any person with a diagnosis for another etiology of liver disease, or alcohol abuse/misuse or alcohol-related liver disease, defined on or prior to the index liver biopsy date.

**Table S2:** Definitions of histological subgroups of MASLD*.

|  | | SNOMED and ICD codes | |
| --- | --- | --- | --- |
| # | Histological subgroup | Inclusion | Exclusion |
| 1 | **Cirrhosis** | M495 [exactly] or M4950x | - |
| 2 | **Noncirrhotic fibrosis (note that this may or may not include MASH)** | Steatosis: either M008x or M5520x, PLUS at least 1 fibrosis code: M49 [exactly], M4900x or M49060. | Cirrhosis codes: M459 [exactly] or M4950x |
| 3 | **MASH without fibrosis** | Steatosis: either M008x or M5520x, PLUS at least one of the following:  1. any M4- code, or 2. M5400x | M4 defines a very broad category of inflammation, both acute and chronic.  Cannot have any of:  Fibrosis codes (M49 [exactly], M4900x or M49060)  OR Cirrhosis: M495 [exactly] or M4950x. |
| 4 | **Simple steatosis** | M5008x or M5520x | Cannot have any:  Inflammation codes: M4- or M5400x  OR Fibrosis: M49 [exactly], M4900x or M49060  OR Cirrhosis: M495 [exactly] or M4950x |

The same table was presented in our previous study.^1^

Abbreviations: MASLD, metabolic dysfunction-associated steatotic liver disease; SNOMED, Systematized Nomenclature of Medicine; ICD, International Classification of Diseases.

*Of note: In Sweden, clinically indicated liver biopsies are generally conducted with a single pass of the liver, unless a satisfactory specimen could not be obtained. According to Swedish liver histopathology reporting recommendations, it is documented if any biopsy is too short in length (i.e., <15 mm in length), has fewer than five portal tracts, or is fragmented, respectively (Svensk Förening för Patologi–Svensk Förening för Klinisk Cytologi. Available at: https://www.svfp.se/foreningar/uploads/L15178/kvast/lever/Leverbiopsier2019.pdf. Accessed April 4, 2019).

**Table S3:** Exclusion of stillbirths.

| Excluded condition | Definition | Data source |
| --- | --- | --- |
| Stillbirth | Death of fetus after 28 completed gestational weeks until July 2008 and thereafter after 22 completed weeks. | *MBR* |

Abbreviations: *MBR, Medical Birth Register.*

**Table S4:** Codes to identify all-cause mortality and cancer in the offspring.

| Outcome | Definition | Data source |
| --- | --- | --- |
| All-cause mortality | Recording between index date and Dec 31, 2021 (end of follow-up in ESPRESSO) | *Cause of Death Register* (primary cause of death) |
| Incident cancer | Recording of corresponding ICD-code between index date and Dec 31, 2021 (end of follow-up in ESPRESSO):   - ICD-10: C00-C99 - ICD-8/ICD-9: 140-209 | *Cancer Register* |

Abbreviations: *MBR*, *Medical Birth Register*

**Table S5:** Definitions of covariates and baseline characteristic variables.

| OFFSPRING BASELINE CHARACTERISTICS AND COVARIATES | | | |
| --- | --- | --- | --- |
| Covariate or baseline characteristic variable | **Categories** | **Data source/s** | **Codes/definitions** |
| Female sex | Male  Female | *Total Population Register* | - |
| Calendar year of delivery (i.e., calendar year of date of birth) | 1990-1999  2000-2010  2011-2017 | *MBR* | - |
| Gestational age at birth [weeks] | <37 weeks  <32 weeks  Missing | *MBR* | - |
| Preterm birth | yes/no | *MBR* | <37 gestational weeks |
| Medically indicated preterm birth | yes/no | *MBR* | Variables in *MBR:* Induced cesarean section, planned cesarean section: ICD-10: O61; ICD-9: 659B. Exclusion of women with premature rupture of the membranes (ICD-10: O42, ICD-9: 658B) |
| Spontaneous preterm birth | yes/no | *MBR* | Variables in *MBR: Spontaneous preterm birth;* premature rupture of the membranes (ICD-10: O42, ICD-9: 658B) |
| Very preterm birth | yes/no | *MBR* | <32 gestational weeks |
| Birth weight [g] | Low  Normal  High  Missing | *MBR* | Low: <2,500  Normal: 2,500 to <4,000  High: ≥4,000  Missing |
| Small for gestational age (SGA) | yes/no | *MBR* | Birth weight <10^th^ percentile below the sex specific mean for gestational age according to the Swedish reference curve. |
| Large for gestational age (LGA) | yes/no | *MBR* | Birth weight >90^th^ percentile above the sex specific mean for gestational age according to the Swedish reference curve. |
| Low birth weight | yes/no | *MBR* | Birth weight <2,500 g (separate for all live births and term live births [gestational age ≥ week 37+0]) |
| Apgar <7 at 5 minutes | yes/no | *MBR* | Apgar <7 (restricted to term live births: ≥37 gestational weeks) |
| Congenital malformations | yes/no | *NPR* and *Cause of Death Register* | ICD-10: Q00–Q99; ICD-9: 740-759 (recordings within 365 days after delivery; restricted to live births) |
| Induction of labor | yes/no | *MBR* | Variable in *MBR*: Induced cesarean section: ICD-10: O61; ICD-9: 659A, 659B |
| Cesarean section | yes/no | *MBR* | Variable in *MBR:* Cesarean section |
| Instrumental delivery | yes/no | *MBR* | Variables in *MBR* |
| MATERNAL BASELINE CHARACTERISTICS AND COVARIATES | | | |
| Covariate or baseline characteristic variable | **Categories** | **Data source/s** | **Codes/definitions** |
| Age at delivery [years] | <25  25-35  ≥35 | *MBR* | - |
| Country of birth | Nordic  Other  Missing | *Total Population Register* | ~~-~~ |
| Parity | Nulliparous (0 pregnancies prior to current pregnancy)  Multiparous (≥1 pregnancies prior to current pregnancy) | *MBR* | - |
| Level of education [years] | ≤9  10-12  ≥13 years  Missing | *LISA* | - |
| BMI in early pregnancy [kg/m^2^] | <18.5  18.5 to <25  25 to <30  ≥30 | *MBR* | - |
| Smoking in early pregnancy | yes/no | *MBR* | **-** |
| Any diabetes (pre-existing type 1 or 2 diabetes or gestational diabetes) | yes/no | *MBR* | Type 1 or 2 diabetes (within 5 years prior to delivery): ICD-10: E10, E11; ICD-9: 250; ICD-8: 250  Gestational diabetes (any time prior to delivery): ICD-10: O24.4; ICD-9: 648W |
| Any hypertension (pre-existing hypertension or gestational hypertension) | yes/no | *MBR* | Hypertension (within 5 years prior to delivery): ICD-10: I10-I16, I13-P; ICD-9: 401, 402, 403, 404, 405; ICD-8: 400-404  Gestational hypertension (any time prior to delivery): ICD-10: O13; ICD-9: 760A |
| Dyslipidemia | yes/no | *NPR* and *PDR* | Within 5 years prior to delivery: ICD-10: E78; ICD-9: 272; ICD-8: 272; ATC: C10AA, C10BA, C10BX, C10AB, C10AC |
| Pre-eclampsia | yes/no | *MBR* and *NPR* | Any time prior to delivery: ICD-10: O14-O15; ICD-9: 642E, 642F, 642G, 642H |

Table adapted from Marxer et al.^1^

Abbreviations: *MBR*, *Medical Birth Register*; *LISA*, Swedish Longitudinal Integrated Database for Health Insurance and Labour Market Studies (*Longitudinell Integrationsdatabas för Sjukförsäkrings- och Arbetsmarknadsstudier*); BMI, body mass index; ICD, International Classification of Diseases; *NPR*, *National Patient Register*; *PDR*, *Prescribed Drug Register*; ATC, Anatomical Therapeutic Chemical; *NPR*, *National Patient Register*.

**Table S6:** Baseline characteristics of A. offspring of women with simple steatosis only vs. matched births in reference women without MASLD, and baseline characteristics of B. offspring of women with severe MASLD (simple steatosis plus MASH without fibrosis, noncirrhotic fibrosis, or cirrhosis) vs. matched reference offspring of women without MASLD.

|  | 1. Simple steatosis | | 1. Severe MASLD* | |
| --- | --- | --- | --- | --- |
|  | Offspring of women with MASLD | Reference offspring | Offspring of women with MASLD | Reference offspring |
| **Offspring, n** | 175 | 833 | 64 | 298 |
| **Unique mothers, n** | 117 | 831 | 44 | 298 |
| **Years of follow-up** |  |  |  |  |
| Median (IQR) | 18.0 [13.3, 23.3] | 18.0 [13.2, 23.5] | 13.3 [9.9, 19.1] | 12.9 [9.2, 18.5] |
| <18 | 88 (50.3) | 423 (50.8) | 46 (71.9) | 221 (74.2) |
| ≥18 | 87 (49.7) | 410 (49.2) | 18 (28.1) | 77 (25.8) |
| **OFFSPRING CHARACTERISTICS** |  |  |  |  |
| **Female sex** | 78 (44.8) | 382 (45.9) | 30 (46.9) | 146 (49.0) |
| **Calendar year of date of birth (i.e., start of follow-up)** |  |  |  |  |
| 1992-1999 | 55 (31.4) | 265 (31.8) | 9 (14.1) | 43 (14.4) |
| 2000-2010 | 89 (50.9) | 426 (51.1) | 36 (56.2) | 169 (56.7) |
| 2011-2017 | 31 (17.7) | 142 (17.0) | 19 (29.7) | 86 (28.9) |
| **Gestational age at birth [days], median (IQR)** | 274.0 [264.5, 283.0] | 280.0 [273.0, 286.0] | 275.0 [264.0, 283.0] | 282.0 [275.0, 288.0] |
| **Preterm birth** |  |  |  |  |
| Any preterm birth (<37 weeks) | 31 (17.7) | 38 (4.6) | 9 (14.1) | 14 (4.7) |
| Medically indicated | 16 (9.1) | 12 (1.4) | 7 (10.9) | 2 (0.7) |
| Spontaneous | 15 (8.6) | 25 (3.0) | 2 (3.1) | 12 (4.0) |
| Very preterm (<32 weeks) | 5 (2.9) | 6 (0.7) | 2 (3.1) | 3 (1.0) |
| **Fetal growth** |  |  |  |  |
| **Birth weight [g]** |  |  |  |  |
| Median (IQR) | 3475 [2963, 3893] | 3595 [3250, 3930] | 3505 [3074, 3903] | 3610 [3235, 3910] |
| Low (<2,500) | 20 (11.4) | 25 (3.0) | 6 (9.4) | 13 (4.4) |
| Normal (2,500 to <4,000) | 121 (69.1) | 630 (75.6) | 45 (70.3) | 223 (74.8) |
| High (≥4,000) | 33 (18.9) | 175 (21.0) | 13 (20.3) | 61 (20.5) |
| Missing | 1 (0.6) | 3 (0.4) | 0 (0.0) | 1 (0.3) |
| **Small for gestational age (SGA)** | 24 (13.8) | 66 (8.0) | 11 (17.2) | 31 (10.4) |
| **Large for gestational age (LGA)** | 32 (18.4) | 103 (12.4) | 16 (25.0) | 33 (11.1) |
| **Apgar <7 at 5 minutes** | 4 (2.3) | 12 (1.5) | 0 (0.0) | 3 (1.0) |
| **Congenital malformations** | 11 (6.3) | 44 (5.3) | 5 (7.8) | 17 (5.7) |
| **Delivered by** |  |  |  |  |
| Induction of labor | 33 (19.6) | 117 (14.2) | 16 (25.0) | 39 (13.1) |
| Cesarean section | 56 (32.0) | 129 (15.5) | 21 (32.8) | 52 (17.4) |
| Instrumental delivery | 10 (5.7) | 55 (6.6) | 5 (7.8) | 27 (9.1) |
| **MATERNAL CHARACTERISTICS** |  |  |  |  |
| **Maternal age at delivery [years]** |  |  |  |  |
| Median (IQR) | 32.0 [28.0, 36.0] | 32.0 [28.0, 36.0] | 30.0 [27.0, 35.0] | 30.0 [27.0, 35.0] |
| 15 to <25 | 13 (7.4) | 61 (7.3) | 13 (20.3) | 62 (20.8) |
| 25 to <35 | 102 (58.3) | 484 (58.1) | 32 (50.0) | 153 (51.3) |
| 35 to 44 | 60 (34.3) | 288 (34.6) | 19 (29.7) | 83 (27.9) |
| **Year of maternal MASLD diagnosis (index liver biopsy)** |  |  |  |  |
| Up until 1999 | 128 (73.1) | - | 33 (51.6) | - |
| 2000-2010 | 43 (24.6) | - | 28 (43.8) | - |
| 2011-2017 | 4 (2.3) | - | 3 (4.7) | - |
| **Disease duration (time between first MASLD diagnosis and delivery [years]** |  |  |  |  |
| Median (IQR) | 5.8 [3.1, 9.9] | - | 5.3 [3.2, 9.9] | - |
| <5 | 75 (42.9) | - | 27 (42.2) | - |
| 5 to <10 | 58 (33.1) | - | 22 (34.4) | - |
| ≥10 | 42 (24.0) | - | 15 (23.4) | - |
| **Maternal country of birth** |  |  |  |  |
| Nordic | 153 (87.4) | 696 (83.6) | 50 (78.1) | 240 (80.5) |
| Other | 22 (12.6) | 137 (16.4) | 14 (21.9) | 58 (19.5) |
| **Civil status of the mother** |  |  |  |  |
| Living with partner | 150 (85.7) | 754 (90.5) | 57 (89.1) | 264 (88.6) |
| Not living with partner | 9 (5.1) | 14 (1.7) | 2 (3.1) | 7 (2.3) |
| Missing | 16 (9.1) | 65 (7.8) | 5 (7.8) | 27 (9.1) |
| **Education** |  |  |  |  |
| Compulsory school (≤9 years) | 26 (14.9) | 83 (10.0) | 10 (15.6) | 33 (11.1) |
| Upper secondary school (10-12 years) | 106 (60.6) | 369 (44.3) | 34 (53.1) | 116 (38.9) |
| College or university (≥13 years) | 43 (24.6) | 366 (43.9) | 20 (31.2) | 142 (47.7) |
| Missing | 0 (0.0) | 15 (1.8) | 0 (0.0) | 7 (2.3) |
| **Parity: multiparous** | 113 (64.6) | 538 (64.6) | 34 (53.1) | 157 (52.7) |
| **BMI in early pregnancy [kg/m²]** |  |  |  |  |
| Median (IQR) | 28.9 [25.0, 33.3] | 24.0 [21.4, 26.8] | 28.0 [25.0, 32.4] | 23.7 [21.6, 27.2] |
| <18.5 | 0 (0.0) | 18 (2.2) | 0 (0.0) | 5 (1.7) |
| 18.5 to <25 | 40 (22.9) | 429 (51.5) | 15 (23.4) | 156 (52.3) |
| 25 to <30 | 49 (28.0) | 202 (24.2) | 23 (35.9) | 69 (23.2) |
| ≥30 | 70 (40.0) | 84 (10.1) | 22 (34.4) | 32 (10.7) |
| Missing | 16 (9.1) | 100 (12.0) | 4 (6.2) | 36 (12.1) |
| **Smoking in early pregnancy** |  |  |  |  |
| Yes | 33 (18.9) | 94 (11.3) | 8 (12.5) | 20 (6.7) |
| No | 135 (77.1) | 695 (83.4) | 52 (81.2) | 264 (88.6) |
| Missing | 7 (4.0) | 44 (5.3) | 4 (6.2) | 14 (4.7) |
| **Prior comorbidities and conditions** |  |  |  |  |
| Diabetes | 12 (6.9) | 9 (1.1) | 13 (20.3) | 1 (0.3) |
| Hypertension | 9 (5.1) | 4 (0.5) | 3 (4.7) | 2 (0.7) |
| Dyslipidemia | 3 (1.7) | 1 (0.1) | 1 (1.6) | 1 (0.3) |
| Pre-eclampsia | 15 (8.6) | 19 (2.3) | 4 (6.2) | 16 (5.4) |

Values are n (%), unless otherwise indicated. Abbreviations: MASLD, metabolic dysfunction-associated steatotic liver disease; n, number; SD, standard deviation; IQR, interquartile range; min, minimum, max, maximum; BMI, body mass index. *Defined as simple steatosis plus MASH without fibrosis, noncirrhotic fibrosis, or cirrhosis. °Diabetes type 1, diabetes type 2, or gestational diabetes. ^+^Including gestational hypertension.

**Table S7:** Baseline characteristics of term born offspring with *in utero* exposure to maternal MASLD and matched reference offspring.

|  | Offspring of mothers with MASLD | Reference offspring |
| --- | --- | --- |
| **Offspring, n** | 199 | 897 |
| **Unique mothers, n** | 138 | 896 |
| **Years of follow-up** |  |  |
| Median (IQR) | 17.5 [12.4, 22.9] | 17.1 [12.0, 22.9] |
| <18 | 108 (54.3) | 502 (56.0) |
| ≥18 | 91 (45.7) | 395 (44.0) |
| **OFFSPRING CHARACTERISTICS** |  |  |
| **Female sex** | 93 (47.0) | 426 (47.5) |
| **Calendar year of date of birth (i.e., start of follow-up)** |  |  |
| 1992-1999 | 56 (28.1) | 260 (29.0) |
| 2000-2010 | 105 (52.8) | 472 (52.6) |
| 2011-2017 | 38 (19.1) | 165 (18.4) |
| **Gestational age at birth [days], median (IQR)** | 278.0 [271.0, 284.0] | 281.0 [275.0, 287.0] |
| **Fetal growth** |  |  |
| **Birth weight [g]** |  |  |
| Median (IQR) | 3590 [3288, 3945] | 3615 [3300, 3940] |
| Low (<2,500) | 5 (2.5) | 9 (1.0) |
| Normal (2,500 to <4,000) | 149 (74.9) | 690 (76.9) |
| High (≥4,000) | 44 (22.1) | 196 (21.9) |
| Missing | 1 (0.5) | 2 (0.2) |
| **Small for gestational age (SGA)** | 25 (12.6) | 67 (7.5) |
| **Large for gestational age (LGA)** | 41 (20.7) | 115 (12.8) |
| **Apgar <7 at 5 minutes** | 1 (0.5) | 5 (0.6) |
| **Congenital malformations** | 9 (4.5) | 43 (4.8) |
| **Delivered by** |  |  |
| Induction of labor | 41 (21.4) | 117 (13.2) |
| Cesarean section | 54 (27.1) | 130 (14.5) |
| Instrumental delivery | 13 (6.5) | 68 (7.6) |
| **MATERNAL CHARACTERISTICS** |  |  |
| **Maternal age at delivery [years]** |  |  |
| Median (IQR) | 32.0 [27.0, 35.0] | 32.0 [27.0, 35.0] |
| 15 to <25 | 25 (12.6) | 109 (12.2) |
| 25 to <35 | 112 (56.3) | 517 (57.6) |
| 35 to 44 | 62 (31.2) | 271 (30.2) |
| **Liver histology of maternal MASLD** |  |  |
| Simple steatosis | 144 (72.4) | - |
| MASH without fibrosis | 27 (13.6) | - |
| Noncirrhotic fibrosis | 24 (12.1) | - |
| Cirrhosis | 4 (2.0) | - |
| **Year of maternal MASLD diagnosis (index liver biopsy)** |  |  |
| Up until 1999 | 137 (68.8) | - |
| 2000-2010 | 56 (28.1) | - |
| 2011-2017 | 6 (3.0) | - |
| **Disease duration (time between first MASLD diagnosis and delivery [years]** |  |  |
| Median (IQR) | 5.4 [2.9, 9.7] | - |
| <5 | 90 (45.2) | - |
| 5 to <10 | 64 (32.2) | - |
| ≥10 | 45 (22.6) | - |
| **Maternal country of birth** |  |  |
| Nordic | 169 (84.9) | 742 (82.7) |
| Other | 30 (15.1) | 155 (17.3) |
| **Civil status of the mother** |  |  |
| Living with partner | 176 (88.4) | 811 (90.4) |
| Not living with partner | 7 (3.5) | 16 (1.8) |
| Missing | 16 (8.0) | 70 (7.8) |
| **Education** |  |  |
| Compulsory school (≤9 years) | 30 (15.1) | 83 (9.3) |
| Upper secondary school (10-12 years) | 115 (57.8) | 394 (43.9) |
| College or university (≥13 years) | 54 (27.1) | 402 (44.8) |
| Missing | 0 (0.0) | 18 (2.0) |
| **Parity: multiparous** | 123 (61.8) | 556 (62.0) |
| **BMI in early pregnancy [kg/m²]** |  |  |
| Median (IQR) | 28.9 [25.1, 33.3] | 23.7 [21.4, 26.8] |
| <18.5 | 0 (0.0) | 16 (1.8) |
| 18.5 to <25 | 44 (22.1) | 471 (52.5) |
| 25 to <30 | 59 (29.6) | 207 (23.1) |
| ≥30 | 78 (39.2) | 96 (10.7) |
| Missing | 18 (9.0) | 107 (11.9) |
| **Smoking in early pregnancy** |  |  |
| Yes | 34 (17.1) | 90 (10.0) |
| No | 158 (79.4) | 768 (85.6) |
| Missing | 7 (3.5) | 39 (4.3) |
| **Prior comorbidities and conditions** |  |  |
| Diabetes* | 16 (8.0) | 8 (0.9) |
| Hypertension° | 9 (4.5) | 3 (0.3) |
| Dyslipidemia | 3 (1.5) | 1 (0.1) |
| Pre-eclampsia | 17 (8.5) | 28 (3.1) |

Values are n (%), unless otherwise indicated.

Abbreviations: MASLD, metabolic dysfunction-associated steatotic liver disease; n, number; SD, standard deviation; IQR, interquartile range; MASH, metabolic dysfunction-associated steatohepatitis; BMI, body mass index.

* Diabetes type 1, diabetes type 2, or gestational diabetes.

° Including gestational hypertension.

**Table S8:** Characteristics of deaths in sensitivity and subgroup analyses.

|  | Offspring of mothers with simple steatosis | | Offspring of mothers with severe MASLD* | | Term born offspring** | | Offspring with normal birth weight for gestational age | | First offspring within a woman | |
| --- | --- | --- | --- | --- | --- | --- | --- | --- | --- | --- |
|  | Offspring of mothers with MASLD | Reference offspring | Offspring of mothers with MASLD | Reference offspring | Offspring of mothers with MASLD | Reference offspring | Offspring of mothers with MASLD | Reference offspring | Offspring of mothers with MASLD | Reference offspring |
| Death during follow-up, n | 2 | 4 | 0 | 3 | 0 | 4 | 0 | 5 | 2 | 5 |
| Age at death [years], mean (SD) | 0.3 (0.3) | 7.8 (10.3) | - | 7.9 (13.5) | - | 13.0 (12.0) | - | 6.2 (9.6) | 0.3 (0.3) | 9.8 (12.3) |

Values are n (%), unless otherwise indicated.

Abbreviations: MASLD, metabolic dysfunction-associated steatotic liver disease; n, number; SD, standard deviation.

*Defined as simple steatosis plus MASH without fibrosis, noncirrhotic fibrosis, or cirrhosis.

**Defined as ≥37 gestational weeks.

**Table S9:** All-cause mortality through early adulthood among offspring with *in utero* exposure to maternal MASLD and matched reference offspring in sensitivity and subgroup analyses.

|  | N | Events | py | IR per 1000 py  (95% CI) | Unadjusted HR  (95% CI)  Model 1* | Adjusted HR  (95% CI)  Model 2** |
| --- | --- | --- | --- | --- | --- | --- |
| Offspring of mothers with simple steatosis |  |  |  |  |  |  |
| Overall | 1008 | 6 | 17973 | 0.3 (0.1-0.7) | - | - |
| Reference offspring | 833 | 4 | 14853 | 0.3 (0.1-0.7) | 1 (Reference) | 1 (Reference) |
| Offspring of mothers with MASLD | 175 | 2 | 3120 | 0.6 (0.1-2.3) | 2.39 (0.44-13.06) | 3.59 (0.48-26.79) |
| Offspring of mothers with severe MASLD° |  |  |  |  |  |  |
| Overall | 362 | 3 | 5076 | 0.6 (0.1-1.7) | - | - |
| Reference offspring | 298 | 3 | 4154 | 0.7 (0.1-2.1) | 1 (Reference) | 1 (Reference) |
| Offspring of mothers with MASLD | 64 | 0 | 922 | 0.0 (0.0-4.0) | NA^+^ | NA^+^ |
| Term born offspring°° |  |  |  |  |  |  |
| Overall | 1096 | 4 | 18849 | 0.2 (0.1-0.5) | - | - |
| Reference offspring | 897 | 4 | 15383 | 0.3 (0.1-0.7) | 1 (Reference) | 1 (Reference) |
| Offspring of mothers with MASLD | 199 | 0 | 3467 | 0.0 (0.0-1.1) | NA^+^ | NA^+^ |
| Offspring with normal birth weight for gestational age |  |  |  |  |  |  |
| Overall | 1076 | 5 | 18184 | 0.3 (0.1-0.6) | - | - |
| Reference offspring | 873 | 5 | 14740 | 0.3 (0.1-0.8) | 1 (Reference) | 1 (Reference) |
| Offspring of mothers with MASLD | 203 | 0 | 3444 | 0.0 (0.0-1.1) | NA^+^ | NA^+^ |
| First offspring within a woman |  |  |  |  |  |  |
| Overall | 927 | 7 | 16194 | 0.4 (0.2-0.9) |  |  |
| Reference offspring | 766 | 5 | 13378 | 0.4 (0.1-0.9) | 1 (Reference) | 1 (Reference) |
| Offspring of mothers with MASLD | 161 | 2 | 2816 | 0.7 (0.1-2.6) | 1.93 (0.37-9.95) | 2.70 (0.34-21.56) |

Abbreviations: py, person-years; IR, incidence rate; CI, confidence interval; HR, hazard ratio; MASLD, metabolic dysfunction-associated steatotic liver disease.

°Defined as simple steatosis plus MASH without fibrosis, noncirrhotic fibrosis, or cirrhosis.

°°Defined as ≥37 gestational weeks.

*Model 1: conditioned on matching set.

**Model 2: conditioned on matching set and further adjusted for maternal obesity-related factors (BMI in early pregnancy, diabetes (including gestational diabetes) any time prior to delivery, and pre-eclampsia any time prior to delivery.

^+^NA: not available (could not be calculated due to lack of deaths among offspring to mothers in this stratum).

**References (Supplementary Material)**

1. Marxer CA, Ebrahimi F, Bergman D, et al. Adverse pregnancy and birth outcomes in women with biopsy-proven MASLD: a nationwide cohort study. *EClinicalMedicine*. Published online May 9, 2025:103238. doi:10.1016/J.ECLINM.2025.103238
